# Effect of maneuvers, diuresis, and fluid administration on ultrasound-measured liver stiffness after Fontan

**DOI:** 10.1097/HC9.0000000000000527

**Published:** 2024-09-18

**Authors:** Pradipta Debnath, Cara E. Morin, Julie Bonn, Samjhana Thapaliya, Clayton A. Smith, Jonathan R. Dillman, Andrew T. Trout

**Affiliations:** 1 Department of Radiology, Cincinnati Children’s Hospital Medical Center, Cincinnati, Ohio, USA; 2 Department of Radiology, University of Cincinnati College of Medicine, Cincinnati, Ohio, USA; 3 Department of Pediatrics, University of Cincinnati College of Medicine, Cincinnati, Ohio, USA; 4 Division of Gastroenterology, Department of Pediatrics, Hepatology and Nutrition, Cincinnati Children’s Hospital Medical Center, Cincinnati, Ohio, USA; 5 Department of Cardiology, Cincinnati Children’s Hospital Heart Institute, University of Cincinnati College of Medicine, Cincinnati, Ohio, USA

## Abstract

**Background:**

To determine the effect of stress maneuvers/interventions on ultrasound liver stiffness measurements (LSMs) in patients with Fontan circulation and healthy controls.

**Methods:**

In this prospective, IRB-approved study of 10 patients after Fontan palliation and 10 healthy controls, ultrasound 2D shear-wave elastography LSMs were acquired at baseline and after maximum inspiration, expiration, standing, handgrip, aerobic exercise, i.v. fluid (500 mL normal saline) administration, and i.v. furosemide (20 mg) administration. Absolute and percent change in LSM were compared between baseline and each maneuver, and then from fluid infusion to after diuresis.

**Results:**

Median ages were 25.5 and 26 years in the post-Fontan and control groups (*p* = 0.796). LSMs after Fontan were higher at baseline (2.6 vs. 1.3 m/s) and with all maneuvers compared to controls (all *p* < 0.001). Changes in LSM with maneuvers, exercise, fluid, or diuresis were not significant when compared to baseline in post-Fontan patients. LSM in controls increased with inspiration (+0.02 m/s, 1.6%, *p* = 0.03), standing (+0.07 m/s, 5.5%, *p* = 0.03), and fluid administration (+0.10 m/s, 7.8%, *p* = 0.002), and decreased 60 minutes after diuretic administration (−0.05 m/s, −3.9%, *p* = 0.01) compared to baseline. LSM after diuretic administration significantly decreased when compared to after i.v. fluid administration at 30 minutes (−0.79 m/s, −26.5%, *p* = 0.004) and 60 minutes (−0.78 m/s, −26.2%, *p* = 0.017) for patients after Fontan and controls at 15 minutes (−0.12 m/s, −8.70%, *p* = 0.002), 30 minutes (−0.15 m/s, −10.9%, *p* = 0.003), and 60 minutes (−0.1 m/s, −10.9%, *p* = 0.005).

**Conclusions:**

LSM after Fontan is higher with more variability compared to controls. Diuresis is associated with significantly decreased liver stiffness in both patients after Fontan and controls, with the suggestion of a greater effect in Fontan patients.

## INTRODUCTION

Measurement and longitudinal monitoring of liver stiffness measurements (LSMs) is emerging as a potential tool for assessing Fontan-associated liver disease (FALD), a common extracardiac complication of Fontan surgical palliation of univentricular cardiac physiology.^1^ LSM immediately increases following Fontan-related cavopulmonary anastomosis,^2^ before the onset of liver fibrosis. In follow-up, LSM remains elevated due to ongoing congestion related to underlying elevated central venous pressure but, with time, may also be increased due to superimposed fibrosis.^1,3–7^

The Society of Radiologists in Ultrasound (SRU) recommends the following patient preparation and imaging technique to obtain LSMs with ultrasound shear-wave elastography: fasting for at least 4 hours, supine or slight left lateral decubitus positioning, and obtaining measurements in a neutral breath hold.^8^ These instructions mitigate factors that affect blood flow to the liver, which may alter the shear-wave elastography and LSM.^9–12^ However, these factors (including the effect of fluid and diuretic administration) have not been robustly studied in patients following Fontan palliation. LSMs may not react similarly to healthy controls with the same constraints, given the altered hemodynamics following Fontan surgery.

The coexistence of congestion and fibrosis following Fontan palliation complicates the interpretation of LSM in these patients. Being able to separate liver stiffening related to congestion from liver stiffening due to fibrosis would have clinical implications for the care of patients after Fontan. Given that the 2 cannot currently be distinguished based on resting LSM, alternative approaches to making this distinction are needed. We hypothesized that provocative maneuvers targeted at altering the hemodynamics of hepatic blood flow might provide additional information that could be subsequently explored in studies seeking to distinguish hepatic congestion from fibrosis. Braun et al^12^ previously showed that fasting and breathing maneuvers (maximum inspiratory and expiratory holds) had no significant effect on liver stiffness measured by ultrasound elastography in patients with Fontan circulation. However, to our knowledge, other methods of dynamically influencing hepatic blood flow hemodynamics have not been explored in patients with Fontan circulation.

Therefore, the objective of this study was to determine the effect of a range of provocative maneuvers, including i.v. fluid administration and diuresis, on LSM by ultrasound shear-wave elastography in patients after Fontan. Furthermore, we sought to compare these measurements to LSM obtained from healthy controls to identify differences in response in post-Fontan patients versus controls.

## METHODS

This prospective, exploratory study was approved by our Institutional Review Board and was compliant with the Health Insurance Portability and Accountability Act. Written informed consent was obtained from all participants.

### Study sample

This study included a matched convenience sample of patients who had previously undergone the Fontan operation (Fontan group) and healthy controls. Ten patients after Fontan and 10 healthy control participants were recruited. Inclusion criteria for participation included age between 18 and 35 years and the ability to tolerate ultrasound scanning of the abdomen. Exclusion criteria included history of heart failure, allergy to furosemide, clinical use of a diuretic medication in the last 30 days, and pregnancy (a pregnancy test was administered to female participants before study participation). All participants were enrolled between February and May 2021. Participants were asked not to eat, drink, or exercise for 4 hours before the study visit.

Participants’ demographic information was retrieved from the electronic medical record or was collected at the study visit. Height and weight were measured upon arrival for the study visit.

### Ultrasound technique

All study-related imaging was obtained on a Canon Aplio i800 ultrasound machine using an i8CX1 transducer. Clinical ultrasound technologists trained in our study protocol performed the ultrasound examinations. Through an intercostal approach, LSMs were taken 1–5 cm deep into the liver capsule at a maximal depth of 7 cm. Five LSMs (in m/s) were obtained at baseline for each maneuver detailed below, after i.v. fluid administration, and at specified time points after i.v. diuretic administration. The medians of each group of 5 measurements were used for statistical analyses.

### Maneuvers and conditions tested

LSMs were obtained in the following order for each study participant: (1) baseline (suspended respiration) obtained in supine positioning by asking the participants to simply stop breathing; (2) supine inspiratory at maximum inhalation breath hold; (3) supine expiratory at maximum exhalation breath hold; (4) Standing after 5 minutes; (5) Trendelenburg with the examination bed positioned at a 15-degree declination with patient in a supine position with feet and legs elevated above the level of the heart for 5 minutes before taking measurements; (6) handgrip exercise after asking the participant to hold a handgrip dynamometer at a calculated 50%–75% of their maximum strength in a supine position while measurement was taken; (7) aerobic exercise-augmented liver stiffness in a supine position after walking for 5 minutes at a normal pace; (8) after i.v. bolus of 10 mL/kg (maximum of 500 mL) 0.9% normal saline infused under gravitational flow over 10 minutes using a 20-gauge needle placed in the upper extremity; and (9) 15, 30, and 60 minutes after i.v. administration of 20 mg furosemide.

Blood pressure, heart rate, and respiratory rate were measured before and after administering i.v. fluid as well as 1 hour after i.v. furosemide administration.

### MRI review

To attempt to stratify patients based on the severity of FALD, 3 board-certified pediatric radiologists (Cara E. Morin, Jonathan R. Dillman, and Andrew T. Trout) reviewed the most recent clinically obtained liver magnetic resonance images (MRI) for the Fontan group (median interval between MRI and study visit: 214 d; range: 3–1136 d) and scored each using the previously described Fontan liver MRI score (based on hepatic congestion, fibrosis, presence of splenomegaly, varices, nodules, and/or ascites).^13^ The MRI examinations were reviewed independently and then together to obtain consensus, as needed. The total score was then calculated for each patient.

### Statistical analysis

Continuous variables were assumed to be non-normally distributed and were summarized as medians and IQRs or ranges.^14^ Categorical variables were summarized as counts and percentages. Paired measurements were compared using the Wilcoxon signed-rank test, and independent measurements were compared with the Mann-Whitney *U* test. Categorical data were compared using the Fisher exact test. Spearman correlation coefficient was carried out to identify linear relationships between variables, and they are presented along with their 95% CIs.

To dichotomize patients for analysis, the median Fontan liver MRI score was calculated, and patients were divided into high Fontan liver MRI scores (above the median) and low Fontan liver MRI scores (below and including the median). We additionally dichotomized based on the median baseline LSM with patients divided into high baseline LSM (above the median) and low baseline LSM (below and including the median).

All statistical analyses were performed using MedCalc Statistical Software (MedCalc Software Ltd.). A *p* value of <0.05 was considered statistically significant for all inference testing.

## RESULTS

### Study sample

Ten patients with Fontan circulation and 10 healthy controls were recruited between February and May 2021. The median ages of the Fontan group and controls were 25.5 (IQR: 23.6–27.9) and 26.0 (IQR: 21.2–31.0) years, respectively (*p* = 0.796). The median time since Fontan operation was 22.2 (IQR: 19.1–24.2) years. Sex, height, weight, and body mass index were similar in both groups (Table 1). Supplemental Table 1, http://links.lww.com/HC9/B39, summarizes the vital signs of patients before and after i.v. fluid administration and after i.v. furosemide administration.

**TABLE 1:** Clinical characteristics of Fontan group and healthy control participants

	Fontan (n = 10)	Control (n = 10)	
Variable	Median (IQR)	Median (IQR)	*p* b
Sex^a^ (F vs. M)	F = 3 (30.0%) M = 7 (70.0%)	F = 5 (50.0%) M = 5 (50.0%)	0.650
Age (y)	25.5 (23.6–27.9)	26.0 (21.2–31.0)	0.796
Height (cm)	167.3 (158.5–174.7)	167.9 (164.6–183.5	0.393
Weight (kg)	70.8 (59.1–81.7)	75.8 (68.6–83.1)	0.393
BMI (kg/m^2^)	24.3 (21.1–28.7)	26.1 (23.2–27.1)	0.796
Years after Fontan	22.2 (19.1–24.2)	NA	NA
Fontan type^a^	Extracardiac conduit = 4 (40.0%) Lateral tunnel = 6 (60.0%)	NA	NA
Fontan liver MRI score^13^	6.0 (2.0–7.0)	NA	NA

^a^ Categorical data are presented as counts and percentages.

^b^ *p* value comparing the Fontan and control groups using the Fisher exact test or Mann-Whitney *U* test as appropriate.

Abbreviations: BMI, body mass index; NA, not applicable.

### LSMs: Fontan versus control groups

**FIGURE 1:**
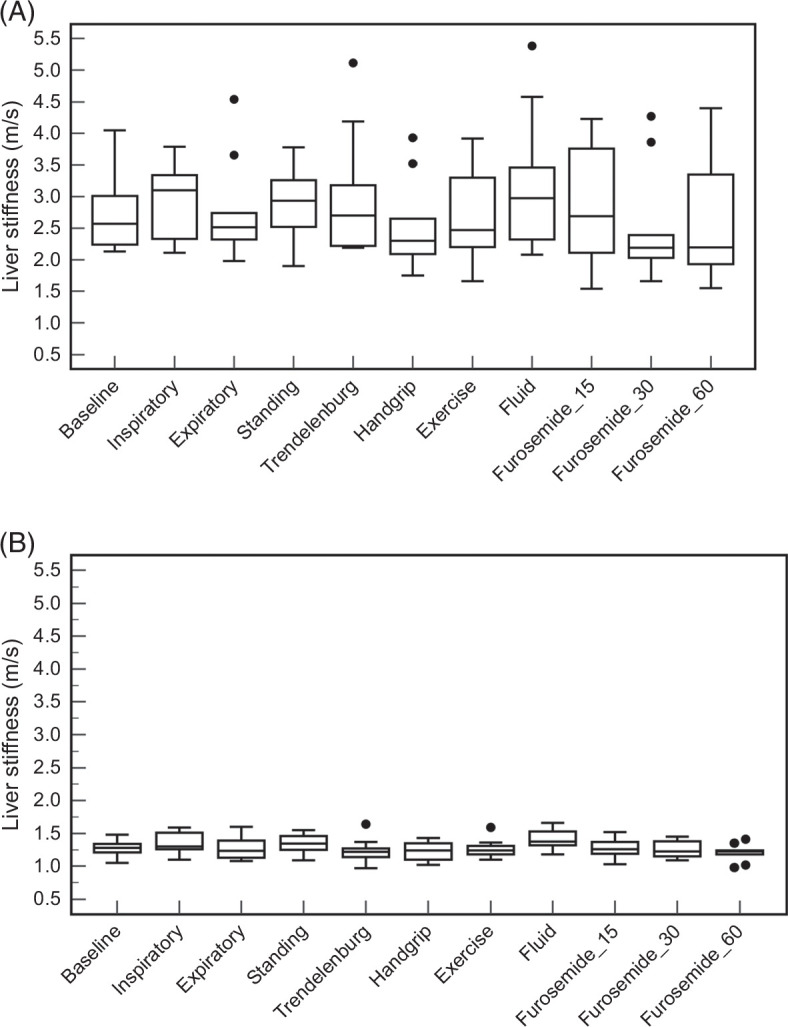
Box and whisker plots of ultrasound shear-wave elastography–measured liver stiffness during various maneuvers, and following fluid and diuretic administration in patients after Fontan (A) and controls (B). Outliers are represented by black circles. Furosemide_15, Furosemide_30, Furosemide_60 represent liver stiffness measurements 15, 30, and 60 minutes after furosemide administration, respectively.

**FIGURE 2:**
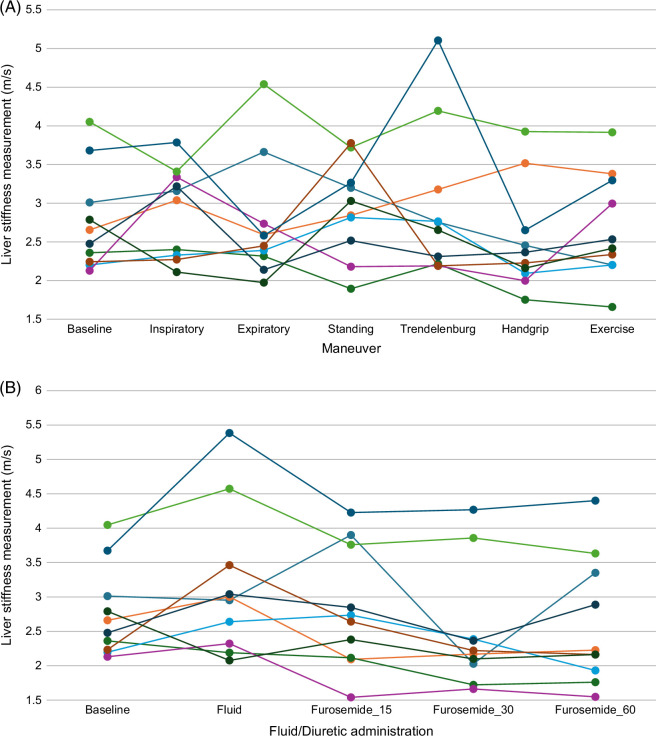
Spaghetti plots of ultrasound shear-wave elastography liver stiffness measurements during (A) various maneuvers and (B) after fluid and diuretic administration in Fontan group patients. Each line represents an individual patient. Furosemide_15, Furosemide_30, and Furosemide_60 represent liver stiffness measurements 15, 30, and 60 minutes after furosemide administration, respectively.

Baseline LSM were significantly higher in the Fontan group than the control group (*p* = 0.0002) (Table 2). Changes in LSM with maneuvers, exercise, fluid, or diuresis were not significant when compared to the baseline in the Fontan group. LSM in controls increased with inspiration (+0.02 m/s, 1.6%, *p* = 0.03), standing (+0.07 m/s, 5.5%, *p* = 0.03), and fluid administration (+0.10 m/s, 7.8%, *p* = 0.002), and decreased 60 minutes after diuretic administration (−0.05 m/s, −3.9%, *p* = 0.01) compared to baseline. The Fontan group had greater variation in LSM with a wider interquartile range compared to the controls for all maneuvers and conditions tested. Figure 1 illustrates the distribution of LSM among the Fontan and control groups for each maneuver and condition tested. To further demonstrate LSM changes for individual patients in the Fontan group, Figure 2 shows LSM for each individual patient across each maneuver and after i.v. fluid and furosemide administration.

**TABLE 2:** Liver stiffness measurements (in m/s), and change relative to baseline, for the Fontan and control groups during maneuvers, exercise, following saline infusion, and diuretics

	Fontan (n = 10)	Change from baseline	Control (n = 10)	Change from baseline	
	Median (IQR)	Absolute (% change from baseline) *p* a	Median (IQR)	Absolute (% change from baseline) *p* a	*p* value between Fontan and control groups^b^
Baseline (suspended respiration)	2.57 (2.24–3.01)	NA	1.28 (1.21–1.34)	NA	**0.0002**
Inspiratory	3.10 (2.33–3.34)	0.53 (20.6%) *p* = 0.232	1.30 (1.26–1.51)	0.02 (1.6%) *p* = **0.028**	**0.0002**
Expiratory	2.52 (2.32–2.74)	−0.05 (−1.94%) *p* > 0.999	1.24 (1.13–1.39)	−0.04 (−3.1%) *p* = 0.959	**0.0002**
Standing	2.94 (2.52–3.26)	0.37 (14.4%) *p* = 0.557	1.35 (1.25–1.46)	0.07 (5.5%) *p* = **0.034**	**<0.0001**
Trendelenburg	2.70 (2.22–3.18)	0.13 (5.1%) *p* = 0.574	1.22 (1.14–1.27)	−0.06 (−4.7%) *p* = 0.074	**0.0002**
Handgrip	2.30 (2.09–2.65)	−0.27 (−10.5%) *p* = 0.059	1.24 (1.10–1.35)	−0.04 (−3.1%) *p* = 0.202	**<0.0001**
Exercise	2.47 (2.20–3.30)	−0.10 (−3.9%) *p* = 0.678	1.24 (1.18–1.31)	−0.04 (−3.2%) *p* = 0.722	**0.0002**
Fluid	2.98 (2.32–3.46)	0.41 (16.0%) *p* = 0.106	1.38 (1.32–1.53)	0.10 (7.8%) *p* = **0.002**	**0.0002**
15 min after furosemide	2.69 (2.11–3.76)	0.12 (4.7%) *p* = 0.946	1.26 (1.19–1.37)	−0.02 (−1.6%) *p* = 0.838	**<0.0001**
30 min after furosemide	2.19 (2.03–2.39)	−0.38 (−14.8%) *p* = 0.083	1.23 (1.15–1.38)	−0.05 (−3.9%) *p* = 0.385	**0.0002**
60 min after furosemide	2.20 (1.93–3.35)	−0.37 (−14.4%) *p* = 0.308	1.23 (1.18–1.24)	−0.05 (−3.9%) *p* = **0.011**	**0.0002**

*Note*: Bold *p* values indicate statistical significance.

^a^ *p* value comparing baseline LSM with LSM following each respective maneuver within each participant group using the Wilcoxon Signed-Rank test.

^b^ *p* value comparing LSM between the Fontan and control groups using the Mann-Whitney *U* test.

Abbreviation: NA, not applicable.

Diuretic administration significantly decreased LSM when compared to LSM obtained following fluid challenge in both groups, with the greatest change ~30 minutes (Fontan group: −26.5%, *p* = 0.04; Control group: −10.9%, *p* = 0.003) after administration (Table 3). Vital signs (blood pressure, heart rate and respiratory rate) before i.v. fluid, after i.v. fluid, and after i.v. diuretic administration are detailed in Supplemental Table 1, http://links.lww.com/HC9/B39 and did not differ between the 2 groups.

**TABLE 3:** Change in LSM (in m/s) after furosemide administration compared to LSMs after fluid infusion in Fontan and control group patients

Group	Time after furosemide	Change in LSM (absolute, % change)	*p* a
Fontan	15 min	−0.29 (−9.73%)	0.202
	30 min	−0.79 (−26.5%)	**0.004**
	60 min	−0.78 (−26.2%)	**0.017**
Control	15 min	−0.12 (−8.70%)	**0.002**
	30 min	−0.15 (−10.9%)	**0.003**
	60 min	−0.15 (−10.9%)	**0.005**

*Note*: Bold text indicates significant *p* values.

^a^ *p* value comparing liver measurement taken after fluid infusion vs after Furosemide administration using the Wilcoxon signed-rank test.

Abbreviation: LSM, liver stiffness measurement.

### LSMs: Fontan group stratified by baseline stiffness

When stratified by baseline LSM, the median LSM in the low and high stiffness groups was 2.24 and 3.01 m/s, respectively (Table 4). There were significant differences in LSM between subgroups at baseline (*p* = 0.008), and with Trendelenburg positioning (low: 2.22 m/s; high: 3.18 m/s; *p* = 0.03), handgrip (low: 2.09 m/s; high: 2.65 m/s; *p* = 0.04), and at 60 minutes after diuretic administration (low: 1.93 m/s; high: 3.35 m/s; *p* = 0.04).

**TABLE 4:** LSM (in m/s) for Fontan group patients with high (above the median shear-wave speed of 2.57 m/s) versus low (below the median shear-wave speed) baseline LSM during maneuvers, exercise, following saline infusion, and diuretics

	Low (<median) Fontan stiffness (n = 5)	Change from baseline	High (>median) Fontan stiffness (n = 5)	Change from baseline	
	Median (IQR)	(absolute, %, change, *p* ^a^)	Median (IQR)	(absolute, % change, *p* ^a^)	*p* b
Baseline (suspended respiration)	2.24 (2.18–2.39)	NA	3.01 (2.76–3.77)	NA	**0.008**
Inspiratory	2.4 (2.32–3.25)	0.16 (7.1%) *p* = 0.063	3.16 (2.81–3.55)	0.15 (5.0%) *p* = 0.813	0.548
Expiratory	2.39 (2.28–2.52)	0.15 (6.7%) *p* = 0.063	2.59 (2.43–3.88)	−0.42 (−14.0%) *p* = 0.625	0.310
Standing	2.52 (2.11–3.06)	0.28 (12.5%) *p* = 0.313	3.2 (2.98–3.38)	0.19 (6.3%) *p* = 0.813	0.151
Trendelenburg	2.22 (2.19–2.43)	−0.02 (−0.9%) *p* > 0.999	3.18 (2.73–4.42)	0.17 (5.6%) *p* = 0.416	**0.028**
Handgrip	2.09 (1.94–2.27)	−0.15 (−6.7%) *p* = **0.042**	2.65 (2.38–3.62)	−0.36 (−12.0%) *p* = 0.438	**0.032**
Exercise	2.34 (2.07–2.65)	0.1 (4.5%) *p* = 0.465	3.3 (2.36–3.52)	0.29 (9.6%) *p* = 0.343	0.143
Fluid	2.64 (2.29–3.15)	0.4 (17.9%) *p* = 0.125	3 (2.73–4.78)	−0.01 (−0.3%) *p* = 0.625	0.548
15 min after furosemide	2.64 (1.97–2.77)	0.4 (17.9%) *p* = 0.813	3.76 (2.31–3.98)	0.75 (24.9%) *p* > 0.999	0.310
30 min after furosemide	2.22 (1.71–2.37)	−0.02 (−0.9%) *p* = 0.313	2.16 (2.08–3.96)	−0.85 (−28.2%) *p* = 0.313	0.548
60 min after furosemide	1.93 (1.71–2.34)	−0.31 (−13.8%) *p* = 0.313	3.35 (2.21–3.83)	0.34 (11.3%) *p* = 0.813	**0.036**

*Note*: Bold text indicates significant *p* values.

^a^ *p* value comparing baseline LSM with LSM following each respective maneuver within each participant group using the Wilcoxon signed-rank test.

^b^ *p* value comparing LSM between the Fontan and control groups using the Mann-Whitney *U* test.

Abbreviation: LSM, liver stiffness measurement.

Patients in the low stiffness subgroup had borderline significant increases in LSM with inspiration (*p* = 0.06) and expiration (*p* = 0.06), as well as a significant decrease in LSM with handgrip (*p*=0.04) (Table 4). For these same maneuvers, there was no significant change in LSM for the patients in the high baseline stiffness subgroup.

### LSMs: Fontan group stratified by fontal liver MRI score

Fontan liver MRI scores ranged from 2 to 9, with a median of 6 (Table 1). There was no statistically significant correlation between the total Fontan liver MRI score and baseline LSM (*r* = −0.09, 95% CI: −0.68 to 0.57, *p* = 0.81). There was no significant difference in LSM with any maneuver or following fluid or diuretic between patients with high versus low Fontan liver MRI scores (Supplemental Table 2, http://links.lww.com/HC9/B39).

## DISCUSSION

Patients who have undergone Fontan palliation of single ventricle congenital heart disease have increased central venous pressure, leading to universal FALD and hepatic fibrosis.^15,16^ Longitudinal assessment of FALD with LSM allows noninvasive liver disease monitoring in combination with laboratory testing. Currently, there is no accurate noninvasive way to separate liver stiffening caused by congestion from that caused by superimposed fibrosis.

In this exploratory study evaluating changes in noninvasive ultrasound LSM with provocative maneuvers, we have shown that at baseline and with all maneuvers, patients in the Fontan group have higher LSM than healthy controls. The Fontan group had considerably more variable LSM than controls and thus showed no statistically significant change in LSM from baseline with provocative maneuvers. Despite the lack of statistical significance, the effect size in the Fontan group was larger in absolute terms than in the control group for the following maneuvers: inspiratory (Fontan: 20.6%, control: 1.6%) standing (Fontan: 14.4%, control: 5.5%), handgrip (Fontan: −10.5%, control: −3.1%), and 60 minutes after Furosemide (Fontan: −14.4%, control: −3.9%).

In addition to group-level differences, our results show greater variability in the LSM response of individual patients in the Fontan group after each maneuver than observed in the control group. This may reflect individual differences in post-Fontan physiology and/or differences in the relative presence of congestion versus fibrosis. Since we did not have liver biopsy data available to help distinguish between the 2, we attempted to explore these differences by stratifying Fontan group patients based on the recently described Fontan liver MRI score^13^ and based on baseline LSM. While we saw no significant difference between Fontan liver MRI subgroups, differences were observed between subgroups when we stratified based on baseline liver stiffness. Specifically, patients in the high baseline stiffness subgroup had higher median LSM in Trendelenburg positioning, with handgrip, and 60 minutes after diuretic administration than patients in the low baseline LSM subgroup. Moreover, patients in the high baseline LSM subgroup seemed to have less change (from baseline) with inspiration, expiration, and handgrip.

Ours is not the first study to explore the impact of provocative maneuvers or changes in fluid status on LSM. Braun et al^12^ previously reported minimal clinical impact of breathing and food intake on LSM measured by ultrasound in patients with normal circulation and no effect in patients with Fontan circulation. Fluid status has been shown to affect LSM in patients with end-stage renal disease, with a significant decrease in LSM after hemodialysis and a strong correlation between liver stiffness and the amount of fluid removed.^17^ We observed a similar effect of decreased LSM following diuretic administration when compared to post-fluid infusion in both groups in our study. These findings suggest that fluid status may be important to consider when performing clinical LSM to decrease variability. Furthermore, these results suggest that diuresis might have a role in reducing congestion at that time of LSM to potentially unmask stiffening related to fibrosis, but further studies are needed to explore this.

Ours was a hypothesis-finding study that was limited in terms of sample size, which impacted our ability to demonstrate statistical significance for differences in LSM, particularly given the observed LSM variability within the Fontan group. Furthermore, we do not have data regarding cardiac function, including central venous pressure, nor do we have cardiac testing or liver biopsy results to correlate with our results. While changes in LSM due to maneuvers were not statistically significant, the absolute and % changes were considerably greater in the Fontan group compared to controls. It is possible that statistically significant differences may have been observed with a larger sample size. The large number of analyses on our relatively small sample size increases the risk of false discovery. Finally, while we used MRI to stratify patients in the Fontan group, the interval between the most recent MRI and the research ultrasound examination was greater than 6 months in half of the participants in the Fontan group. This very likely impacted the strength of associations observed.

In conclusion, patients after Fontan have higher LSM, greater variability in LSM, and appear to show greater changes in LSM with provocative maneuvers than controls. There are also differences between individual post-Fontan patients in their response to provocative maneuvers and fluid status, which may reflect differences in underlying cardiac and Fontan physiology as well as differences in the degree of liver congestion and/or fibrosis. The observed differences between groups and between patient subgroups, particularly with Trendelenburg positioning, handgrip, and 60 minutes after diuretic administration, deserve further exploration as potential methods of characterizing FALD. Our results also suggest that fluid status should be considered when measuring LSM in patients after Fontan as it can have a considerable impact on LSM.

## Supplementary Material
