# Supplementary material for: Effect of maneuvers, diuresis, and fluid administration on ultrasound-measured liver stiffness after Fontan
Source: Hepatol Commun. 2024 Sep 18;8(10):e0527. doi: 10.1097/HC9.0000000000000527 (PMC11412719; doi:10.1097/HC9.0000000000000527)
Supplement: Supplementary file 1 [file hc9-8-e0527-s001.docx]

**Supplemental Table 1:** Vital signs for Fontan and control group patients before fluid, after fluid and after Furosemide.

| Variables | Time period | Fontan (n=10) | Control (n=10) | P value* |
| --- | --- | --- | --- | --- |
|  |  | Median  (IQR) | Median  (IQR) |  |
| Systolic Blood Pressure  (mm Hg) | Before fluid | 118.5  (111.0 to 132.0) | 115.5  (110.0 to 121.0) | 0.384 |
|  | After fluid | 112.5  (107.0 to 122.0) | 113.000  (107.0 to 117.0) | 0.677 |
|  | After Furosemide | 117.0  (110.0 to 120.0) | 113.5  (106.0 to 115.0) | 0.210 |
| Diastolic Blood Pressure  (mm Hg) | Before fluid | 74.5  (60.0 to 81.0) | 67.0  (65.0 to 73.0) | 0.650 |
|  | After fluid | 70.0  (63.0 to 76.0) | 62.5  (56.0 to 72.0) | 0.650 |
|  | After Furosemide | 73.5  (62.0 to 79.0) | 74.500  (71.0 to 78.0) | 0.545 |
| Heart rate  (per minute) | Before fluid | 63.0  (51.0 to 74.0) | 63.000  (60.0 to 66.0) | 0.762 |
|  | After fluid | 56.5  (51.0 to 71.0) | 67.500  (60.0 to 70.0) | 0.495 |
|  | After Furosemide | 62.5  (54.0 to 78.0) | 62.500  (56.0 to 72.0) | 0.940 |
| Respiratory rate  (per minute) | Before fluid | 17.0  (16.0 to 18.0) | 16.0  (14.0 to 18.0) | 0.485 |
|  | After fluid | 16.0  (14.0 to 19.0) | 15.0  (14.0 to 20.0) | 0.729 |
|  | After Furosemide | 16.5  (16.0 to 18.0) | 16.000  (14.0 to 16.0) | 0.241 |

IQR: Interquartile range

* p-value comparing Fontan and control groups using Mann-Whitney U test.

**Supplemental Table 2.** Ultrasound shear-wave elastography liver stiffness measurements for Fontan group patients with high (above the median score of 6) versus low (below or equal to the median) Fontan liver MRI scores (13) during maneuvers, exercise, and following saline infusion and diuretic.

|  | Low (<median) Fontan score on MRI (n=7) | Change from baseline | High (>median) Fontan score on MRI (n=3) | Change from baseline | P value * |
| --- | --- | --- | --- | --- | --- |
|  | Median  (IQR) | (absolute, %, change, p value§) | Median  (IQR) | (absolute, % change, †) |  |
| Baseline (Suspended respiration) | 2.66  (2.27 to 2.96) | N/A | 2.48  (2.22 to 3.66) | N/A | >0.999 |
| Inspiratory | 2.40  (2.29 to 3.13) | -0.26  (-9.8%)  p=0.297 | 3.34  (3.25 to 3.39) | 0.86  (34.7%) | 0.117 |
| Expiratory | 2.45  (2.34 to 2.59) | -0.21  (-7.9%)  p=0.813 | 2.74  (2.29 to 4.09) | 0.26  (10.5%) | 0.517 |
| Standing | 3.03  (2.83 to 3.25) | 0.37  (13.9%)  p=0.469 | 2.52  (2.27 to 3.42) | 0.04  (1.6%) | 0.667 |
| Trendelenburg | 2.75  (2.33 to 3.08) | 0.09  (3.38%)  p=0.498 | 2.31  (2.22 to 3.72) | -0.17  (-6.9%) | 0.648 |
| Handgrip | 2.23  (2.11 to 2.60) | -0.43  (-16.2%)  p=0.219 | 2.37  (2.09 to 3.54) | -0.11  (-4.4%) | 0.833 |
| Exercise | 2.34  (2.20 to 3.08) | -0.32  (-12.0%)  p=0.344 | 3.00  (2.65 to 3.69) | 0.52  (21.0%) | 0.137 |
| Fluid | 2.95  (2.30 to 3.35) | 0.29  (10.9%)  p=0.375 | 3.04  (2.50 to 4.20) | 0.56  (22.6%) | 0.667 |
| 15 minutes after Furosemide | 2.64  (2.18 to 3.61) | -0.02  (-0.8%)  p=0.57 | 2.85  (1.87 to 3.53) | 0.37  (14.9%) | >0.999 |
| 30 minutes after Furosemide | 2.16  (2.05 to 2.35) | -0.5  (-18.8%)  p=0.219 | 2.36  (1.84 to 3.49) | -0.12  (-4.8%) | >0.999 |
| 60 minutes after Furosemide | 2.16  (1.99 to 3.07) | -0.5  (-28.8%)  p=0.578 | 2.89  (1.89 to 3.45) | 0.41  (16.5%) | 0.909 |

IQR=interquartile range

§ p-value comparing baseline LSM with LSM following each respective maneuver within each participant group using Wilcoxon Signed-Rank test.

†Cannot calculate p-values using Wilcoxon Signed-Rank test due to small sample size (n=3).

*p-value comparing LSM between the Fontan and control groups using Mann-Whitney U test.
